# Globalization and Health

**DOI:** 10.1186/1744-8603-1-1

**Published:** 2005-04-22

**Authors:** Greg Martin

**Affiliations:** 1 London School of Hygiene and Tropical Medicine, 1 Keppel Street, London, WC1E 7HT, UK

## Abstract

This debut editorial of *Globalization and Health *introduces the journal, briefly delineating its goals and objectives and outlines its scope of subject matter. 'Open Access' publishing is expected to become an increasingly important format for peer reviewed academic journals and that *Globalization and Health *is 'Open Access' is appropriate. The rationale behind starting a journal dedicated to globalization and health is three fold:

Firstly: Globalization is reshaping the social geography within which we might strive to create health or prevent disease. The determinants of health – be they a SARS virus or a predilection for fatty foods – have joined us in our global mobility. Driven by economic liberalization and changing technologies, the phenomenon of 'access' is likely to dominate to an increasing extent the unfolding experience of human disease and wellbeing.

Secondly: Understanding globalization as a subject matter itself needs certain benchmarks and barometers of its successes and failings. Health is one such barometer. It is a marker of social infrastructure and social welfare and as such can be used to either sound an alarm or give a victory cheer as our interconnectedness hurts and heals the populations we serve.

And lastly: In as much as globalization can have an effect on health, it is also true that health and disease has an effect on globalization as exemplified by the existence of quarantine laws and the devastating economic effects of the AIDS pandemic.

A balanced view would propose that the effects of globalization on health (and health systems) are neither universally good nor bad, but rather context specific. If the dialogue pertaining to globalization is to be directed or biased in any direction, then it must be this: *that we consider the poor first*.

## 

I am pleased to introduce '*Globalization and Health*', a peer reviewed, open access (free to the end user) journal. In this, the début editorial, I will briefly outline the purpose and scope of this journal highlighting our intention to publish a balanced mixture of opinion on the subject.

That the journal be 'Open Access' is entirely appropriate. Knowledge, at its best utility, is a 'public good' i.e. non-rival, non-excludable. While this journal will deal with the subject matter of creating 'global public goods for health', it will also by virtue of its very existence, contribute toward that process. *Globalization and Health's *'Open Access' policy changes the way in which articles are published. First, all articles become freely and universally accessible online, and so an author's work can be read by anyone at no cost. Second, the authors hold copyright for their work and grant anyone the right to reproduce and disseminate the article, provided that it is correctly cited and no errors are introduced [1]. Third, a copy of the full text of each Open Access article is permanently archived in an online repository separate from the journal. *Globalization and Health's *articles are archived in PubMed Central [2], the US National Library of Medicine's full-text repository of life science literature, and also in repositories at the University of Potsdam [3] in Germany, at INIST [4] in France and in e-Depot [5], the National Library of the Netherlands' digital archive of all electronic publications. Importantly, the results of publicly funded research will be accessible to all taxpayers and not just those with access to a library with a subscription. As such, Open Access could help to increase public interest in, and support of, research. Note that this public accessibility may become a legal requirement in the USA if the proposed Public Access to Science Act is made law [6]. Added to this, a country's economy will not influence its scientists' ability to access articles because resource-poor countries (and institutions) will be able to read the same material as wealthier ones (although creating access to the internet is another matter [7]).

The rationale behind starting a journal dedicated to globalization and health is three fold:

Firstly: Globalization is reshaping the social geography within which we might strive to create health or prevent disease. The determinants of health – be they a SARS virus or a predilection for fatty foods – have joined us in our global mobility. Driven by economic liberalization and changing technologies, the phenomenon of 'access' is likely to dominate to an increasing extent the unfolding experience of human disease and wellbeing.

Secondly: Understanding globalization as a subject matter itself needs certain benchmarks and barometers of its successes and failings. Health is one such barometer. It is a marker of social infrastructure and social welfare and as such can be used to either sound an alarm or give a victory cheer as our interconnectedness hurts and heals the populations we serve.

And lastly: In as much as globalization can have an effect on health, it is also true that health and disease has an effect on globalization as exemplified by the existence of quarantine laws and the devastating economic effects of the AIDS pandemic.

A balanced view would propose that the effects of globalization on health (and health systems) are neither universally good nor bad, but rather context specific. The extent to which individual states are able to engage the process of globalization on their own terms differs widely from one country to the next. Child mortality, for example, changes quickly in response to subtle changes in purchasing power in impoverished communities. In affluent communities however, a small change in income has little effect on utility in either direction. As we consider the effects of globalization on wellbeing it becomes apparent that we need to consider both the long term scenarios for populations as a whole, and the immediate effects for the more vulnerable within those populations who are dependent on fragile local economies.

If the dialogue pertaining to globalization is to be directed or biased in any direction, then it must be this: *that we consider the poor first*.

## Competing interests

The author(s) declare that they have no competing interests.
